# Increased antitumor efficacy of PD-1-deficient melanoma-specific human lymphocytes

**DOI:** 10.1136/jitc-2019-000311

**Published:** 2020-01-29

**Authors:** Lucine Marotte, Sylvain Simon, Virginie Vignard, Emilie Dupre, Malika Gantier, Jonathan Cruard, Jean-Baptiste Alberge, Melanie Hussong, Cecile Deleine, Jean-Marie Heslan, Jonathan Shaffer, Tiffany Beauvais, Joelle Gaschet, Emmanuel Scotet, Delphine Fradin, Anne Jarry, Tuan Nguyen, Nathalie Labarriere

**Affiliations:** 1 Université de Nantes, Inserm, CRCINA, F-44000 Nantes, France; 2 LabEx IGO, Université de Nantes, Nantes, France; 3 Université de Nantes, Inserm, CRTI, F-44000 Nantes, France; 4 NGS Assay Research & Development, Qiagen Sciences, Frederick, Maryland, United States

**Keywords:** CD8-Positive T-Lymphocytes, cell engineering, costimulatory and inhibitory T-cell receptors, immunotherapy, adoptive, melanoma

## Abstract

**Background:**

Genome editing offers unique perspectives for optimizing the functional properties of T cells for adoptive cell transfer purposes. So far, *PDCD1* editing has been successfully tested mainly in chimeric antigen receptor T (CAR-T) cells and human primary T cells. Nonetheless, for patients with solid tumors, the adoptive transfer of effector memory T cells specific for tumor antigens remains a relevant option, and the use of high avidity T cells deficient for programmed cell death-1 (PD-1) expression is susceptible to improve the therapeutic benefit of these treatments.

**Methods:**

Here we used the transfection of CAS9/sgRNA ribonucleoproteic complexes to edit *PDCD1* gene in human effector memory CD8^+^ T cells specific for the melanoma antigen Melan-A. We cloned edited T cell populations and validated *PDCD1* editing through sequencing and cytometry in each T cell clone, together with T-cell receptor (TCR) chain’s sequencing. We also performed whole transcriptomic analyses on wild-type (WT) and edited T cell clones. Finally, we documented in vitro and in vivo through adoptive transfer in NOD scid gamma (NSG) mice, the antitumor properties of WT and PD-1KO T cell clones, expressing the same TCR.

**Results:**

Here we demonstrated the feasibility to edit *PDCD1* gene in human effector memory melanoma-specific T lymphocytes. We showed that PD-1 expression was dramatically reduced or totally absent on *PDCD1*-edited T cell clones. Extensive characterization of a panel of T cell clones expressing the same TCR and exhibiting similar functional avidity demonstrated superior antitumor reactivity against a PD-L1 expressing melanoma cell line. Transcriptomic analysis revealed a downregulation of genes involved in proliferation and DNA replication in PD-1-deficient T cell clones, whereas genes involved in metabolism and cell signaling were upregulated. Finally, we documented the superior ability of PD-1-deficient T cells to significantly delay the growth of a PD-L1 expressing human melanoma tumor in an NSG mouse model.

**Conclusion:**

The use of such lymphocytes for adoptive cell transfer purposes, associated with other approaches modulating the tumor microenvironment, would be a promising alternative to improve immunotherapy efficacy in solid tumors.

## Background

Among the growing arsenal of therapeutic strategies, adoptive cell transfer (ACT) of natural or engineered tumor-specific T cells still represents a relevant option, notably for immunogenic tumors such as melanoma. Adoptive transfer of tumor infiltrating lymphocytes (TILs) has a long clinical history and provided proofs of efficiency in an adjuvant setting, with extremely long relapse-free survival for some treated patients^1^ and also for curative treatment of metastatic melanoma, when associated with prior lymphodepletion.^2^ These approaches are continually evolving toward an increased specificity and improved tumor reactivity of infused T cells that can be reached either with the use of genetically modified T cells, such as TCR^3–5^ and chimeric antigen receptor (CAR)-transduced T cells^6 7^ or with cloned or sorted T cells specific for selected human leucocyte antigen (HLA)-peptide complexes.^8–12^ The clinical efficacy of these approaches can be further improved by the combination of ACT with tumor-specific T cells endowed with optimized functions (eg, improved avidity of infused T cells) and the blockade of immunomodulatory checkpoints, such as PD-1. Indeed, tumor-reactive T lymphocytes express high levels of PD-1 following antigen encounter and effective activation.^13 14^ This high PD-1 expression renders them susceptible to programmed death ligands (PD-L)1/PD-L2-mediated inhibition in the tumor microenvironment (TME). The combination of cellular therapies with immunotherapies blocking the PD-1 axis is on the rise in preclinical studies and in clinical trials. Several clinical trials are currently evaluating the efficiency of the adoptive transfer of TIL in combination with anti-PD-1/PD-L1-specific antibodies, in melanoma and other solid tumors (clinicaltrial.gov). A critical point related to the combination of ACT and systemic immune checkpoint blockade (ICB) is the occurrence of adverse events due to ICB-related toxicities and autoimmunity. In this context, favoring PD-1 blockade directly within the TME through the infusion of genetically modified tumor-specific T cells could be a relevant alternative strategy to reduce these adverse events, while preserving the therapeutic efficacy. These T cells could be either knocked-out for the expression of PD-1 or engineered to produce single-chain variable fragment (ScFv) forms of anti-PD-1 antibody. This latter strategy has been successfully validated in CD19 CART cells, demonstrating that the local production of anti-PD-1 ScFv prevented CAR T cells from inhibition and exhaustion and improved their ability to eradicate established tumors, contrary to systemic injection of anti-PD-1 antibody.^15^ Alternatively, editing *PDCD1* gene, using the CRISPR/Cas9 technology, in high avidity tumor-specific T cells prior to ACT also appears a promising approach.

The CRISPR/Cas9 system has emerged as a very specific and simple tool for genome editing either for gene knock-out or for the addition or correction of specific gene mutations.^16^ The successful use of CRISPR technology was first demonstrated in human primary T cells with the silencing of CCR5 gene in HIV-1-susceptible human CD4^+^ T cells.^17^ Thus, the CRISPR/Cas9 genome editing system provides an unprecedented and promising technological breakthrough to modify selected human T cell subsets and improve the antitumor efficiency of ACT treatments.^18^ PD-1 inactivation using CRISPR/Cas9 editing has been first reported in human primary T cells^19^ and later in CAR-T cells targeting CD19,^20^ hepatocellular carcinoma^21^ and more recently mesothelin in breast cancer.^22^ In all cases, engineered CAR T cells exhibited enhanced tumor control in mouse models. Improved effector functions have also been reported following *PDCD1* gene editing in virus-specific cytotoxic T lymphocytes (CTL)^23 24^ and in myeloma-specific CTL.^25^ In melanoma, the superior antitumor efficacy of *PDCD1*-edited human-specific CTL has not been formally documented so far. In this study, we demonstrated the feasibility of *PDCD1* gene editing in high avidity effector T cells, specific for the Melan-A antigen, using electroporation of ribonucleic complexes. We further derived and fully characterized *PDCD1*-edited T cell clones and demonstrated their superior antitumor activity in comparison with their wild-type counterparts, in NSG mice engrafted with PD-L1 expressing human melanoma tumors.

## Methods

### Cell lines and cell culture

Sorting and amplification of Melan-A specific T cells was performed as previously described.^12^ Melan-A specific T cell clones were derived from *PDCD1*-edited populations by limiting dilution, as previously described, and clonality was assessed by sequencing TCR alpha and beta chains.^26^ CD8^+^ lymphocytes were cultured in RPMI1064 medium supplemented with 8% human serum, 2 mM L-glutamine (Gibco, France), 100 U/mL penicillin (Gibco), 0.1 mg/mL streptomycin (Gibco) and 150 U/mL human recombinant IL-2 (Proleukin, Novartis Pharma, France). T cell lines and T cell clones were regularly (every 3 weeks) amplified on irradiated feeder cells and PHA-L (1 µg/mL, Sigma, France).

The human transporter associated with antigen processing (TAP)-deficient cell line T2 (174 × CEM.T2) used as a presenting cell was purchased from the ATCC (CRL-1992). The melanoma cell line M113, registered in the Biocollection PC-U892-NL (CHU Nantes, France), was established from metastatic tumor fragments in the Unit of Cell Therapy of Nantes. The T2 cell line and melanoma cell line M113 were also transfected with a eukaryotic expression vector (pCDNA3) bearing human *PDCD1LG1* gene (NM 14143.2, Sino Biological, HG10084-UT) in order to express human PD-L1. The melanoma cell lines M113 or M113^PD-L1+^ and the human TAP-deficient cell lines T2 and T2 ^PD-L1+^ were culture in RPMI1640 medium supplemented with 10% fetal bovine serum (Eurobio), 2 mM L-glutamine (Gibco), 100 U/mL penicillin (Gibco) and 0.1 mg/mL streptomycin (Gibco). M113 melanoma cell line and the T2 cell line expressing PD-L1 were also supplemented respectively with 0.8 mg/mL and 0.45 mg/mL of G418 antibiotic.

All cells were cultured at 37°C in a humidified atmosphere containing 5% CO_2_, and a weekly test was performed through a HEK-Blue Detection Kit (hb-det3, InvivoGen) to check the absence of mycoplasma contamination.

### Electroporation of CAS9/sgRNA complexes in Melan-A-specific CTL lines

Melan-A-specific CTL lines were activated 3 days with immobilized anti-CD3 antibody (400 ng/mL) (OKT3 clone, CRL-8001, ATCC). Prior to the electroporation, T lymphocytes were washed twice in serum-free medium (Optimem, Gibco, France). The sgRNA (0.45 µM) targeting the first exon of *PDCD1* (5′-CGACTGGCCAGGGCGCCTGTGGG-3′)^27^ was denatured at 80°C for 2 min and kept on ice for 2 min before being complexed with CAS9 protein (0.3 µM final) (produced by TACGENE platform CNRS UMR 7196/INSERM U1154) for 10 min at room temperature. These complexes were added to 10^6^ T lymphocytes, in 100 µL of serum-free medium, to which was added the HDR template at 100 pmoles/µL,^27^ in electroporation vials. The electroporation program used was for poring pulse: voltage 225 V; pulse length 5 ms; pulse interval 50 ms; number of pulses 2; decay rate 10%; polarity**+** and for transfer pulse: voltage 20 V; pulse length 50 ms; pulse interval 50 ms; number of pulses 5; decay rate 40% and polarity ± (Nepa21, Nepagene, France). Electroporated T lymphocytes were then recovered in complete medium with 150 U/mL of interleukin (IL)-2, during 48 hours at 37°C, before amplification or cloning on feeder cells.

### Allele modification and off-target analysis

The genomic DNA from T cells was purified using the QIAamp DNA Mini Kit (Qiagen, USA) from 2×10^6^ T cells. The T7 Endonuclease1 assay was performed for detection of the NHEJ repair or HDR (for *PDCD1* gene). The DNA fragment spanning the gene-editing target sites was amplified by PCR from the genomic DNA using the primer pairs indicated in online supplementary table S1. The PCR product was denatured and reannealed in a thermocycler with the following steps: 95°C, 5 min; 95°C–85°C at −2°C/s; 85°C–25°C at −0.1°C/s; hold at 4°C. Then, 10 µL (100–250 ng) of the denaturated–reannealed PCR fragments spanning the gene-editing target sites were mixed with to 2.5 µL of NEB 10× buffer2 and 5 U of T7 Endonuclease I (NEB, USA) in a final volume of 25 µL. Subsequently, the reaction mixture was incubated for 15 min at 37°C. PCR and T7E1 products were run on 5K chip in capillary electrophoresis device (Caliper Labchip-GX, LifeScience). Fluorescence signal ratio of the WT bands to the cut bands gave the Cutting index. Amplified fragments were also sequenced using the primers forward (5′-ACAGTTTCCCTTCCGCTCAC-3′) and reverse (5′-CCGACCCCACCTACCTAAGA-3′) by Eurofins (France). The analysis of each sequence chromatogram was carried out using APE software. Overlapping sequences due to DNA editing were depicted manually to identify indels.

10.1136/jitc-2019-000311.supp1 Supplementary data

An exhaustive list of potential off-targets (OT) with 1–4 mismatches was provided by the CRISPOR software with predicted scores.^28^ For *PDCD1*, nine high scored OT PCR were designed, and gDNAs were analyzed with the T7EI assay. The DNA fragments spanning the potential off-target sites were amplified by PCR from the genomic DNA using the primer pairs listed in online supplementary table S1.

### TCR sequencing

Total RNA from 2×10^6^ T cells was extracted from antigen-specific T cell clones using NucleoSpin RNA II kit (Macherey-Nagel, Germany). For reverse transcriptions, PCR amplifications and sequencing were performed as described,^29 30^ and we have followed throughout the manuscript the IMGT TCR nomenclature.^31^

### Expression of costimulation molecules and immune checkpoints (IC) at rest and after stimulation

CD8^+^ T lymphocytes were activated for 12 hours in 96-well plates coated with anti-CD3 Ab (OKT3 clone, CRL-8001, ATCC) at different concentrations ranging from 50 mg/mL to 400 ng/mL. Immune checkpoints (ICs) expression was determined by labeling with anti-PD-1 (Clone EH12, BD Biosciences), anti-TIGIT (Clone A15153G, BioLegend), anti-LAG-3 (Clone 11C3C65, BD Biosciences), anti-Tim-3 (Clone F38-2E2, Biolegend) and anti-KLRG1 (Clone 14C2A07, Biolegend) antibodies. An anti-CD25 (clone M-A251, BD Biosciences) specific-antibody was also used as a T cell activation marker. The expression of the main costimulation molecules was assessed on resting T lymphocytes with anti-CD45RO (clone UCHL1, BD Biosciences), anti-CD28 (Clone CD28.2, BD Biosciences), anti-CD27 (Clone L128, BD Biosciences), anti-CD2 (Clone RPA-2.10, BD Biosciences) and anti-LFA-1 (Clone HI111, BD Biosciences) monoclonal antibodies. All the cytometric analyses were performed on a Facs Canto II (BD Biosciences).

### qPCR for *PDCD1* gene expression

Total RNA was extracted from activated wild type (WT)and knock-out (KO) T cell clones using NucleoSpin RNA II kit (Macherey-Nagel, France). One microgram of total RNA was reverse-transcribed using SuperScript III reverse transcriptase and oligodT (Thermo Fisher Scientific, France). Relative quantification of *PDCD1* and housekeeping genes *RPLPO* and *Cyclophilin-A* was performed using brilliant SYBR Green qPCR with an Mx4000 machine (Agilent Technologies). *PDCD1*-specific primers were purchased from Qiagen (catalog number PPH13086G). *RPLPO*-specific forward and reverse primer sequences were respectively: 5′-GTGATGTGCAGCTGATCAAGACT-3′ and 5′-GATGACCAGCCCAAAGGAGA-3′. Cyclophilin-A specific forward and reverse primer sequences were respectively: 5′-CCACCGTGTTCTTCGACAT-3′ and 5′-CCAGTGCTCAGAGCACGAAA-3′. Thermal cycling was one step at 95**°**C for 10 min, followed by 40 cycles at 95**°**C for 30 s and 60**°**C for 1 min. Relative *PDCD1* expression was calculated from duplicate values with the 2-ΔΔCt method, using WT4 gene expression values, as references.

### Functional avidity of Melan-A-specific T lymphocytes

Functional avidity of WT and *PDCD1*-edited Melan-A-specific T cell clones was evaluated after coculture with TAP-deﬁcient T2 cells loaded with a range of Melan-A_A27L_ (ELAGIGILTV) peptide at the effector/target ratio 1/2, through the measurement of CD107a mobilization and cytokine production. CD107a mobilization was measured after 3 hours of coculture at 37°C in the presence of a CD107a-speciﬁc mAb (H4A3 clone, BioLegend). T lymphocytes were then stained with anti-CD8 antibodies (Clone RPA-T8, BioLegend) and analyzed by flow cytometry. Cytokine production was determined after a 5-hour stimulation period at 37°C, in the presence of Brefeldin A at 10 µg/mL (Sigma, B7651). T cell clones were labeled with anti-CD8 mAb (Clone RPA-T8, BioLegend), ﬁxed with PBS 4% paraformaldehyde (VWR, 100504-858) and stained for cytokine production using anti-tumor necrosis factor (TNF)-α (Clone MAB11, BioLegend), anti-interferon (IFN)-γ (Clone B27, BioLegend) and anti-IL2 (Clone MQ1-17H12, BioLegend) mAbs. All the cytometric analyses were performed on a Facs Canto II (BD Biosciences).

### Melanoma cell line characterization

The melanoma cell lines M113 and M113^PD-L1+^ (2×10^5^ cells) were labelled with anti-PD-L1 (clone MIH1, BD Biosciences), HLA-A2 (clone BB7.2, BD Biosciences), ICAM-1 (CD54, clone HA58, BD Biosciences) and LFA-3 (CD58, clone 1C3, BD Biosciences) monoclonal antibodies (mAbs). All the antibodies were phycoerythrin (PE) conjugated. For Melan-A intracellular staining, cells were fixed with PBS 4% paraformaldehyde (VWR, 100504-858), permeabilized, incubated with Melan-A-specific mAb (clone A103, Dako, Denmark A103) antibodies, and stained with PE-conjugated goat Fab’2 antimouse IgG secondary Ab (Beckman Coulter, France). All the cytometric analyses were performed on a FACS Canto II (BD Biosciences).

### Reactivity against peptide-loaded T2 cell lines and melanoma cell lines

The reactivity of T cell clones was measured after coculture at 37°C with the T2 cell lines loaded with the Melan-A_A27L_ peptide, at indicated concentrations, at an effector/target ratio of 1/2, and the HLA-A2 melanoma cell lines M113 and M113^PD-L1+^ at different effector/target ratios (1/0.5, 1/1, 1/2 and 1/3). CD107a mobilization was measured after 3 hours of coculture at 37°C in the presence of mAb speciﬁc for CD107a (H4A3 clone, BioLegend). T clones were then labelled with an anti-CD8 antibody (Clone RPA-T8, BioLegend) and analyzed by flow cytometry.

The production of IFN-γ and IL-2 cytokines was determined on supernatants by IFN-γ-specific ELISA (Invitrogen, France) and IL-2-specific ELISA (e-biosciences, France) after 12 hours of stimulation at 37°C, with peptide-loaded T2 cell lines or melanoma cell lines, according to the manufacturer’s recommendations.

### Western blotting analysis

Whole cell lysates were prepared from T cell clones at rest or after stimulation by an anti-CD3 antibody (OKT3 clone, CRL-8001, ATCC) at 400 ng/µL overnight. Briefly, T cells were washed with cold PBS and recovered in lysis buffer (10 mM KCL, 1,5 mM MgCl_2_, 10 mM Tris-HCl pH 7.7, 0.5 mM DTT (0.5 mM)) supplemented with EDTA-free Protease Inhibitor Cocktail (1X, Sigma), Na_3_VO_4_ (Sodium Orthovanadate, Sigma) and passed through the needle (25G). The concentration of proteins was determined by the BCA method (Sigma Aldrich).

Fifteen micrograms of proteins were separated on a 12% SDS-polyacrylamide electrophoresis gel and transferred onto a PVDF membrane (GE Healthcare, LifeScience) in Tris-glycine buffer (Tris 25 mM, glycine 200 mM, methanol 20%) for 90 min at 100 V. The membrane was saturated in a TBS-T buffer (TBS (Tertbutyldimethylsilyl) 1X, Tween-20 0.1%) containing 5% milk for a minimum duration of 1 hour at room temperature. The primary antibodies (polyclonal anti-PD-1 antibody (Abcam), and monoclonal antitubulin antibody, B-5-1-2 (Santa Cruz Biotechnology)) were incubated on the membrane overnight at 4°C in TBST-T 5% milk. Then, the membrane was incubated for 1 hour at room temperature with secondary antibody (HRP-conjugated goat antimouse antibody (Jackson Immunology Research)). The revelation was achieved by adding ECL (‘Enhanced ChemiLuminescent’) reagent (Clarity, Western ECL Substrate, Bio-Rad) via an image acquisition station (Chemi-Doc MP Imager Station, BioRad).

### 3’ transcriptome sequencing

QIAseq UPX 3′ Transcriptome libraries (Qiagen, USA) were prepared with 10 ng of total human RNA from resting and OKT-3-activated T cell clones according to the manufacturer’s handbook. Biologic triplicates (for activated T cell clones) were analyzed in two independent sequencing runs. Briefly, during the reverse transcription reaction, using an anchored oligo-dT primer, each sample was tagged with a unique identifier and each molecule was tagged with a unique molecular index (UMI). Following reverse transcription that also incorporated template switching, all of the individually tagged cDNAs were combined enabling preceding library construction steps to be executed in a single tube. The amplified DNA is fragmented, end repaired and A-tailed within a single, controlled multienzyme reaction. The fragmentation reaction was as follows: 4°C for 1 min; 32°C for 5 min; 65°C for 30 min; hold at 4°C. The DNA fragments were then ready to be ligated at their 5′ ends to a sequencing platform-specific adapter. A universal library amplification step introduces a single sample index and ensures that the DNA fragments containing the cell ID and UMI are sufficiently amplified for NGS.

The yield of the final libraries was determined using Qiagen’s QIAseq Library Quant Array Kit per manufacturer’s recommendations (https://www.qiagen.com/us/products/human-id-and-forensics/nextgeneration-sequencing/qiaseq-library-quant-system/%23resources). Quality control was performed by capillary electrophoresis on a TapeStation System (Agilent Technologies) using a High Sensitivity D5000 Screen Tape.

Each library was diluted and normalized to 4 nM according to the QIAseq Library Quant Array Kit results prior to pooling equally and denaturing. The denatured library pool with a final concentration of 1.2 pM was run on a NextSeq High Output V2.5 kit (Illumina) with 15% PhiX spiked in using paired end sequencing with Read 1 being 100 cycles and Read 2 being 50 cycles.

### Transcriptomic analyses

Normalization and differential expression analysis were performed using the DESeq2^32^ (R-package, version: 1.24.0). Samples and genes were filtered before the analysis. Low-quality samples (UMI <50 000 and genes <5000) were removed (19/96). After that, genes having less than 100 UMI or more than 3 null values across all samples were filtered out (23923/30143). We then had a count matrix for 6220 genes across 77 samples.

Samples were then tested for differential expression, considering the batch effect between the two sequencing runs, p values were corrected using the Benjamini-Hochberg procedure. We selected the 50 most significant genes with an absolute log2 fold change greater than 1 between PD-1^KO^ and WT TRBV3-1 clones. The same procedure was applied to compare KO6 and WT4 clones.

Gene expression values were then transformed (using the vst function from DESEQ2, log2(n+1)) and normalized (using DESEQ2 median of ratios method) for IC plotting. Batch effect regression was performed using the removeBatchEffect function from the limma R package (version: 3.40.2).^33^ Values were further mean centred for heatmap plots. Statistical analysis for IC expression was performed with a Mann-Whitney non-parametric unpaired U test.

### Mouse xenograft model

Female NSG mice aged 6–8 weeks, purchased from Charles River laboratory, with unrestricted access to food and water, were kept under speciﬁc pathogen-free conditions in the UTE animal facility (SFR François Bonamy, IRS-UN, University of Nantes, license number: B-44–278). Subcutaneous xenograft tumors were established by injections of 7×10^5^ of human melanoma cells (M113 or M113^PD-L1+^) in 100 µL DPBS, into the flank of NSG mice. At day 8 (when tumor volumes reached around 100 mm^3^), tumor-bearing mice were randomly allocated into three groups and assigned to receive one of the following intravenous injections: (1) sterile DPBS, (2) 5×10^6^ WT cell clones in sterile DPBS and (3) 5×10^6^ PD-1^KO^ T cell clones in sterile DPBS. Intravenous injections were repeated twice at days 15 and 22 after engraftment. Tumor burdens were measured by an electronic calliper, and the tumor volume was calculated based on the following formula: volume=L × W × W/2, where L was length and W was width.^34^ Mice were sacrificed taking into account the appearance of necrosis in tumors, weight loss (20% of initial weight before tumor transplantation) and tumor size (>2000 mm^3^), in accordance with national and international policies. Tumors were removed at the time of sacrifice. Statistical analyses were performed using two-way analysis of variance multiple comparisons.

### Immunohistochemistry

Tumors were collected, formalin fixed and paraffin embedded. Immunohistochemistry was performed on 3 µm paraffin sections of each tumor using anti-PD-L1 (10 µg/mL, clone E1L3N, Cell Signaling, USA) or anti-CD3 (6 µg/mL, polyclonal, Agilent, USA) primary antibodies, followed by the Peroxidase/DAB Envision detection system (Agilent) on an automated platform (Dako Autosatiner), according to manufacturer’s instructions. The sections were counterstained with Mayer’s hematoxylin. As negative control, omission of the primary antibody was performed. CD3^+^ cells were quantified on whole tissue sections, both in intratumoral areas and peritumoral stroma, with the open source software Qupath, using automatic classification and positive detection workflows.^35^ Results are expressed as the percentage of CD3+ cells relative to the total number of cells.

## Results

### PDCD1 editing in human Melan-A-specific CTL lines

**Figure 1:**
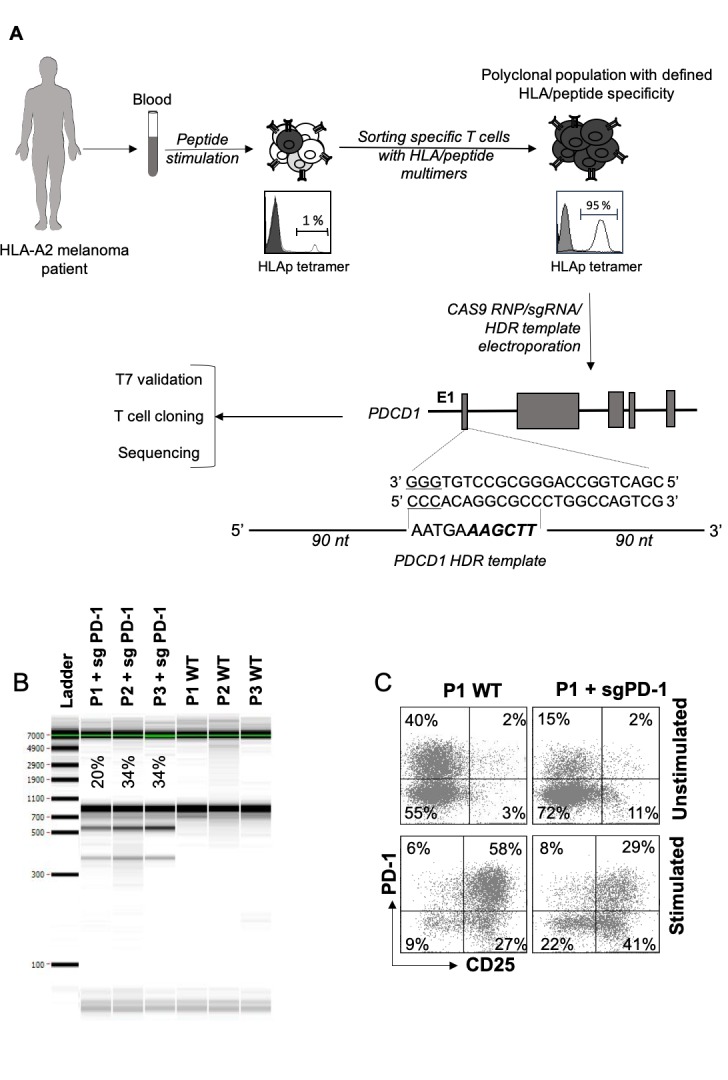
CRISPR/Cas9 editing in melanoma specific CD8^+^ T cells. (A) Production process of *PDCD1*-edited Melan-A-specific T cell clones. Melan-A-specific polyclonal CD8^+^ T lymphocytes were derived from PBMC from HLA-A2 melanoma patients, after a step of peptide stimulation followed by a step of T cell sorting with HLA-peptide coated magnetic beads.^12^ Specific polyclonal T cells were electroporated with Cas9 protein, together with a sgRNA specific for the first exon of *PDCD1* and an HDR template.^26^ The sequence targeted by the sgRNA is indicated, with PAM sequences underlined, as well as the single-stranded oligonucleotide HDR template, with 90-nt homology arms, designed to replace 12 nt, including the PAM sequence, and introduce an HindIII restriction site (bold, italics). T7E1 assay was used to estimate the % of total edition (sum of NHEJ and HDR events that generate indels at the Cas9 cleavage site) in each polyclonal population. Sequencing of *PDCD1* targeted region was further performed on individual T cell clones. (B) Detection by PCR and T7E1 cleavage assay of sgRNA/CAS9-mediated edition of *PDCD1* gene in 3 Melan-A-specific CTL lines, electroporated or not. Percentages of indels are indicated for each edited T cell population. (C) Representative example of the expression pattern of PD-1, by a Melan-A-specific CTL population, edited (right) or not (left) on *PDCD1* gene. PD-1 expression was measured at rest (upper panel) or after anti-CD3 activation (lower panel). The activation status was validated by CD25 labeling Cas9, CRISPR associated protein 9; CRISPR, clustered regularly interspaced short palindromic repeats.

We generated Melan-A specific CTL lines from the blood of HLA-A2 melanoma patients, according to a procedure already described^12^ and currently used to produce melanoma-specific CTL in a clinical trial (ClinicalTrials.gov Identifier: NCT02424916). Briefly, Melan-A-specific CTL lines were specifically sorted with HLA-peptide multimer-coated magnetic beads, after a step of peptide stimulation of patients’ PBMC. These effector memory polyclonal T cell lines are fully specific and reactive against HLA-A2 melanoma cell lines (figure 1A). Three polyclonal T cell populations were further electroporated with ribonucleoprotein complexes (CAS9-sgRNA-HDR template), targeting a region located in the first exon of *PDCD1* gene.^27^ The HDR template was added to enhance *PDCD1* editing, as previously described.^27^ As illustrated by figure 1B, the editing of *PDCD1* gene was effective in these three T cell populations, with 20%–34% of heteroduplexes generated following T7 endonuclease assay. Further experiments were carried out with the P1 edited T cell population. First of all, we controlled the potential off-target effects of the selected sgRNA, which exhibited a high score of specificity for the target sequence and low off-target numbers, as predicted by the CRISPOR software.^28^ An exhaustive list of off-target genes (OT), with 1–4 mismatches, was provided by the CRISPOR software with scores for predicted OT (online supplementary table S1). Nine high scored OT PCR primers were designed, and gDNA from WT and *PDCD1*-edited T cell populations was analyzed by T7 endonuclease assay and sequencing. None of these nine OTs were affected by the selected sgRNA in this *PDCD1*-edited T cell population (online supplementary table S1). Indeed, no heteroduplexes were detected for OT-2, 5, 16, 18, 19 and 30, and although we detected heteroduplexes for OT-1, 3 and 5, they were present in both WT and *PDCD1*-edited T lymphocytes, and not at the expected size, suggesting a non-specific event.

We then evaluated the expression of PD-1 on the P1-edited population compared with the original population, at rest (figure 1C, upper panel) and after 12 hours of anti-CD3 stimulation (figure 1C lower panel). The fraction of PD-1 expressing T cells was decreased by about half in the edited-T cell population in both conditions confirming the T7 assay results.

We thus cloned this population by limiting dilution in order to obtain WT and PD-1^KO^ T cell clones.

### Characterization of wild-type and PDCD1-edited T cell clones

We obtained 14 Melan-A specific T cell clones, 5 wild-type and 9 PD-1^KO^ T cell clones. Sequencing of CDR3 alpha and beta chains of each clone (online supplementary table S2) revealed that seven T cell clones (two WT and five PD-1^KO^) shared the same TCR (TRBV3-1), suggesting that this T cell clonotype was over-represented in the original T cell population. We also determined the recombination event that occurred on *PDCD1* gene in the edited T cell clones, by sequencing a region of 160 bp, encompassing the sgRNA sequence. For the nine edited T cell clones, the recombination event occurred on a single *PDCD1* allele, illustrated by the lower DNA sequencing chromatogram on online supplementary figure S1A. A deletion of 53 nt was observed in three T cell clones (KO2, KO6 and KO11) sharing the same TCR (TRBV3-1), resulting in the loss of PD-1 initiation codon (online supplementary figure S1B and table S2). The two other T cell clones expressing this same TCR (KO1 and KO5) presented either a single nucleotide deletion or a 7 nt deletion, both resulting in a frameshift and a premature stop-codon at the end of the first exon. Three other T cell clones also shared the same TCR (TRBV6-2). Among them, two exhibited the same deletion of 10 nt in *PDCD1* gene, resulting in a frameshift and a premature stop-codon at the end of the first exon. The third of these clonotypes was the only one for which we detected the insertion of the HDR template, resulting in a frameshift and a premature stop codon at the end of the first exon. Finally, the T cell clone KO10 (TRBV28) underwent a deletion of 25 nt resulting in the loss of the initiation codon.

10.1136/jitc-2019-000311.supp2 Supplementary data

**Figure 2:**
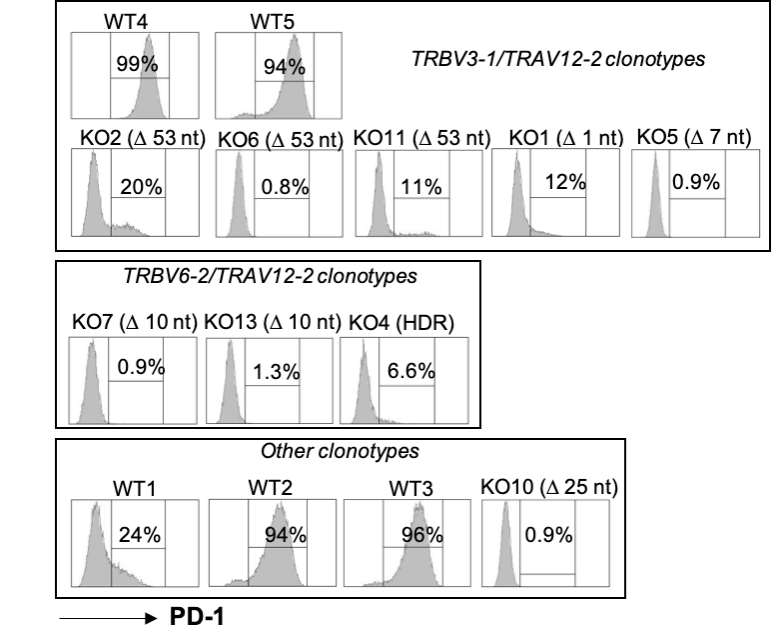
PD-1 expression on *PDCD1*-edited T cell clones. PD-1 expression was analyzed by flow cytometry after activation of T cell clones with anti-CD3 antibody (400 ng/mL, 12 hours). T cell clones were grouped according to their clonotypes. Between brackets is indicated the event on *PDCD1* gene.

All the WT T cell clones, except WT1, highly expressed PD-1 after activation (figure 2). Concerning *PDCD1*-edited T cell clones, we observed either a total absence of PD-1 expression for five T cell clones, independently of their clonotype or the event on *PDCD1* gene (KO5, KO6, KO7, KO10 and KO13), or a strongly reduced expression of PD-1 for four T cell clones (KO1, KO2, KO4 and KO11). This residual expression in these four PD-1^KO^ T cell clones results probably from the expression of the wild-type allele. Conversely, for the negative PD-1^KO^ T cell clones, we can hypothesize that the expression of the wild-type allele was insufficient to lead to the membrane expression of PD-1 molecule. For further experiments, we focused on the TRBV3-1 T cell clones that included both wild-type and PD-1^KO^ T cell clones to evaluate the functional consequences of *PDCD1* editing in melanoma-specific T cell clones.

### PDCD1 expression in TRBV3-1 clonotypes

**Figure 3:**
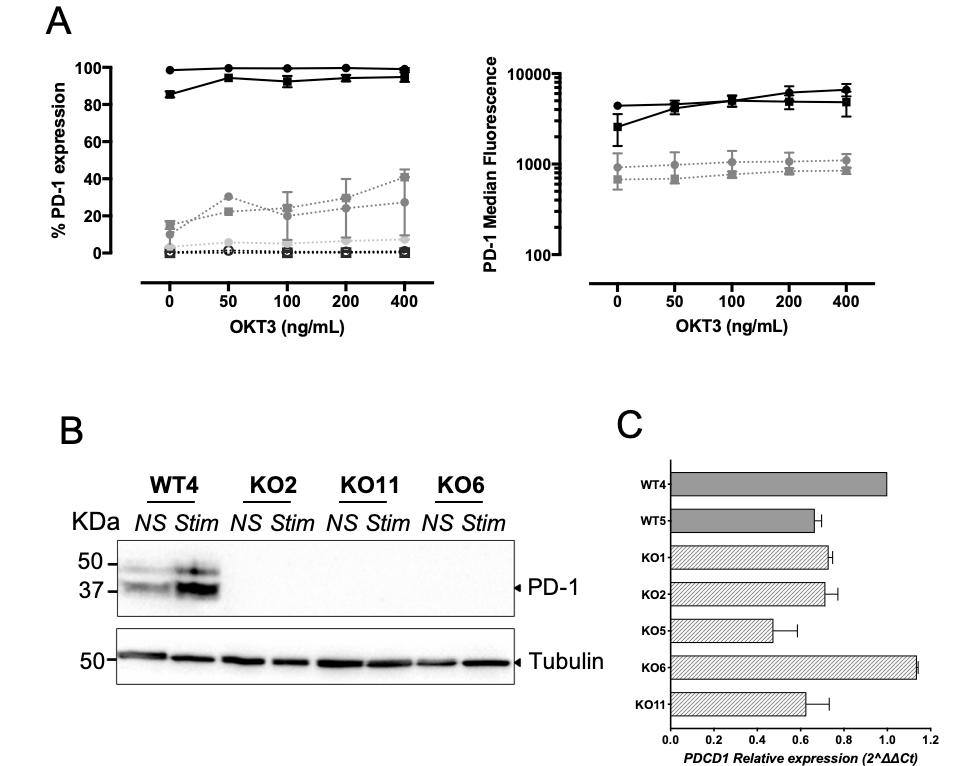
PD-1 expression in TRBV3-1 clonotypes. (A) The fraction (left) and the median of fluorescence (right) of PD-1 expressing T cells were measured on the seven TRBV3-1 clonotypes. PD-1 expression was measured after 12 hours of activation with coated anti-CD3 (OKT3, n=3) antibody at the indicated concentrations. Solid and dotted lines, respectively, illustrate PD-1 expression on wild-type (black circles: WT4; black squares: WT5) and *PDCD1*-edited (dark gray circles: KO1; dark gray squares: KO2; light gray circles: KO11; empty circles: KO5; empty squares: KO6) T cell clones. (B) Western blot analysis of PD-1 expression of resting (NS) and OKT3-activated (STIM) T cell clones. Tubulin was used as a loading control. (C) *PDCD1* relative expression was measured by RT-qPCR on WT T cell clones (gray bars) or *PDCD1*-edited T cell clones (hatched bars) after 12 hours of activation with OKT3. Presented values are calculated with the 2-ΔΔCt method, normalized on WT4 *PDCD1* expression.

We first confirmed the PD-1 expression profiles of the seven TRBV3-1 T cell clones (two WT and five PD-1^KO^ T cell clones), after stimulation with a range of concentrations of anti-CD3 antibody (figure 3A). Indeed, WT cell clones strongly expressed PD-1 (even at rest), whereas PD-1 expression could only be observed on a small fraction (30%–40%, left panel) of two PD-1^KO^ T cell clones (KO1 and KO2), and with a much lower intensity than on the two wild-type T cell clones (figure 3A, right panel). PD-1 expression was not detected on the two other PD-1^KO^ T cell clones (KO5 and KO6), at rest and after stimulation. This absence of PD-1 expression on KO6 T cell clone was further confirmed by western blot (figure 3B). Of note, PD-1 expression was not detected either in KO2 and KO11 T cell clones by western blot, suggesting that the small fraction of positive cells detected by flow cytometry, combined with the low intensity of PD-1 expression, prevents the detection of PD-1 protein from a lysate of these two PD-1^KO^ T cell clones. Finally, we checked by RT-qPCR that *PDCD1* gene expression was not affected by gene editing, and we did not observe any significant differences between WT and PD-1^KO^ T cell clones, concerning *PDCD1* expression (figure 3C).

### Functional avidities of TRBV3-1 T cell clones and comparison of a selected pair of WT/edited T cell clones

**Figure 4:**
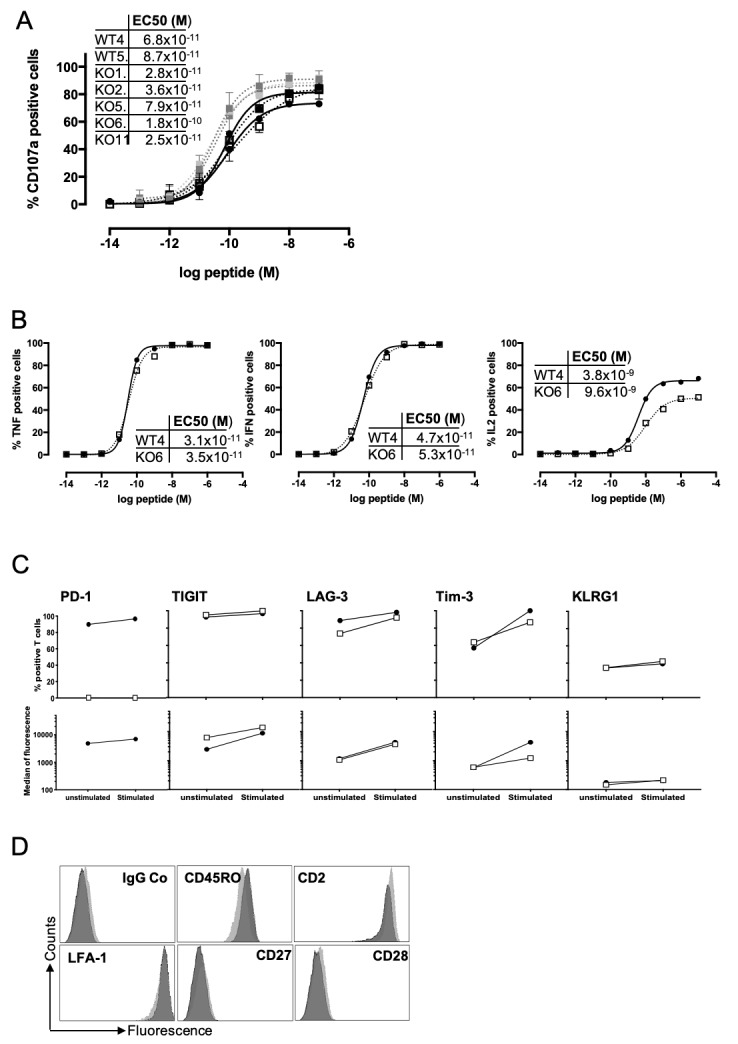
Functional avidity of TRBV3-1 clonotypes. (A) Functional avidities of *PDCD1*-edited (dotted lines) and wild-type TRBV3-1 T cell clones (solid lines) were evaluated by measuring CD107a membrane expression in response to T2 cells loaded with a range of Melan-A_A27L_ peptide, at an E:T ratio of 1:2. CD107a membrane expression was evaluated by double staining with anti-CD8 and anti-CD107a monoclonal antibodies. Table illustrates the EC50 of peptide concentration for each tested T cell clone. (B) Functional avidities of WT4 (black circles, solid lines) and KO6 T cell clones (empty squares, dotted lines) were evaluated by measuring TNF-α, IFN-γ and IL-2 expression in response to T2 cells loaded with a range of Melan-A_A27L_ peptide, at an E:T ratio of 1:2, in presence of brefeldin A. Cytokine expression was evaluated by double staining with anti-CD8 and anticytokine monoclonal antibodies. Tables illustrate the EC50 of peptide concentration for each tested function. (C) Expression of ICP by WT4 and KO6 T cell clones. The fraction (upper panel) and the median of fluorescence (lower panel) of ICP expressing T cells were measured at rest and after 12 hours of activation with coated anti-CD3 antibody at 400 ng/mL. Black circles and white squares, respectively, illustrate ICP expression on WT4 and KO6 T cell clones. (D) Expression of the main costimulatory molecules by WT4 (dark gray histograms) and KO6 (light gray histograms) T cell clones, measured by flow cytometry, at rest.

A prerequisite for in vitro and in vivo comparisons of WT and PD-1^KO^ T cell clones was to ensure that the T cell clones used had comparable functional avidities. Indeed, although the seven selected T cell clones expressed the same TCR, subtle differences in the expression of costimulation molecules could lead to variations in their global functional avidity. We thus measured the functional avidity, based on CD107a mobilization, of the seven TRBV3-1 T cell clones in response to TAP-deficient T2 cells loaded with a range of the Melan-A peptide A27L (figure 4A). The seven tested T cell clones exhibited similar functional avidity ranging from 1.8×10^–10^M to 2.5×10^–11^M. We thus selected a pair of T cell clones (WT4 and KO6) for further experiments, because of their similar functional avidity and because of the total absence of PD-1 expression by KO6 T cell clone, on activation. As for the polyclonal population, we checked the absence of cleavage of the nine predicted off-target genes in KO6 T cell clone (data not shown), and we further confirmed the similar functional avidity of these two T cell clones on the basis of cytokine production (TNF-α, IFN-γ and IL-2). As illustrated by figure 4B, these two T cell clones exhibited very comparable functional avidity measured by TNF-α and IFN-γ production (figure 4B left and middle panel). Their functional avidity, measured by IL-2 production (figure 4B, right panel) was also similar, nevertheless the maximal fraction of T cells producing IL-2 was lower for the KO6 T cell clone (around 50%) than for the WT4 T cell clone (around 65%). We next measured the expression of additional inhibitory molecules at rest and after anti-CD3 stimulation on these two T cell clones (figure 4C), as well as the expression of the main costimulatory molecules at rest (figure 4D). Considering ICP expression, we observed an increased expression level of TIGIT on KO6 T cell clone at rest and after stimulation, and a decreased expression of Tim-3 in stimulated KO6 T cell clone (both in terms of % of positive T cells and expression level). The expression of LAG-3 and KLRG1 were similar in the two T cell-clones, despite a slightly smaller fraction of LAG-3 positive T cells for KO6 T cell clone, at rest. Finally, the two T cell clones exhibited a similar expression profile for the costimulatory molecules tested, illustrating their status of differentiated effector memory T cells (figure 4D).

### Tumor reactivity of WT and PD-1^KO^ T cell clones on PD-L1 expressing cell lines

**Figure 5:**
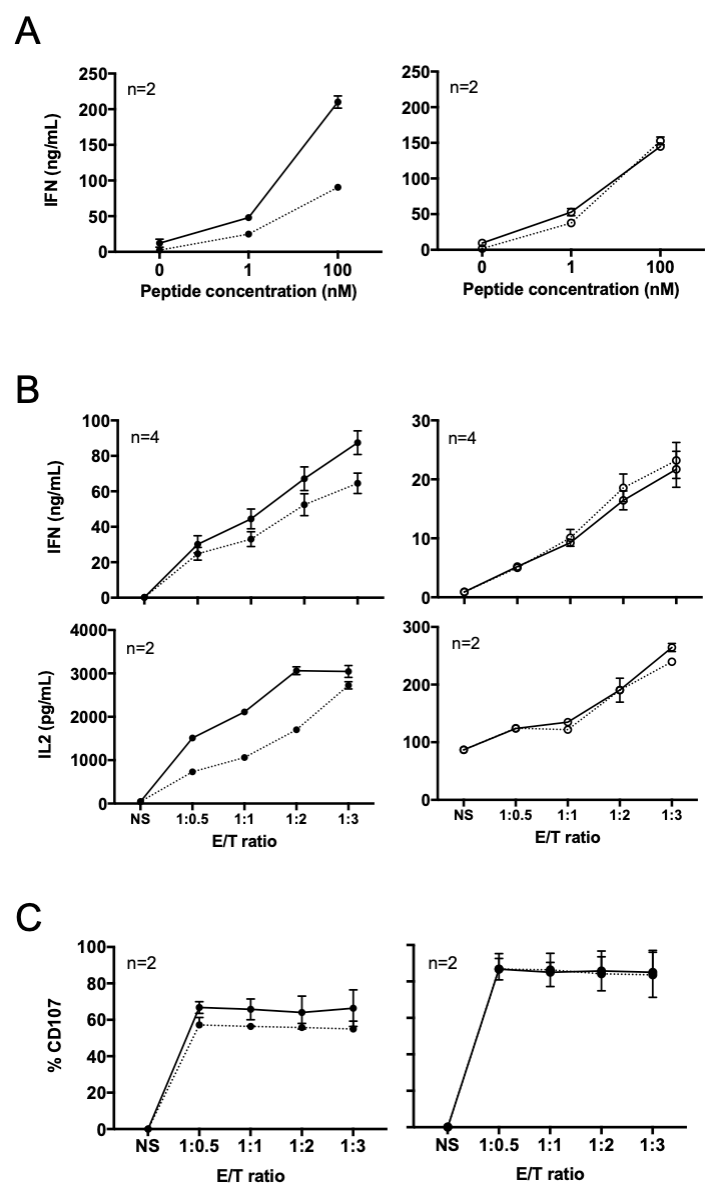
Reactivity against PD-L1 expressing T2 cells and melanoma cells. (A) IFN-γ production of WT4 (left) and KO6 (right) T cell clones in response to peptide-loaded T2 cell lines. IFN-γ production was measured by ELISA in supernatants of T cell clones after 12 hours of activation with wild-type T2 cell line (black or empty circles, solid lines) or T2^PD-L1+^ cell line (black or empty circles, dotted lines), loaded with different concentrations of Melan-A_A27L_ peptide. The number of biologic replicates is indicated in each figure. (B) IFN-γand IL-2 production of WT4 (left) and KO6 (right) T cell clones in response to melanoma cell lines. IFN-γproduction was measured by ELISA in supernatants of T cell clones after 12 hours of activation with M113 melanoma cell line (black or empty circles, solid lines) or M113^PD-L1+^ melanoma cell line (black or empty circles, dotted lines) at the indicated E/T ratios. The number of biologic replicates is indicated in each figure. (C) CD107a membrane expression of WT4 (right) and KO6 (left) T cell clones after 3 hours of activation with M113 melanoma cell line (black or empty circles, solid lines) or M113^PD-L1+^ melanoma cell line (black or empty circles, dotted lines), at the indicated E/T ratios. The number of biologic replicates is indicated in each figure.

We compared the reactivity of PD-1-edited and WT T cell clones on the Tap-deficient T2 cell line (stably expressing or not PD-L1) loaded with the Melan-A_A27L_ antigenic peptide, or on a HLA-A2 melanoma cell line expressing or not PD-L1. As the T2 cell line and cultured human melanoma cell lines do not express spontaneously PD-L1, we previously established these PD-L1 expressing cell lines through stable transfection of a PD-L1 expression plasmid.^36^ The reactivity of T cell clones was tested on the PD-L1-positive cell lines and their non-transfected counterparts by IFN-γ and IL-2 specific ELISA tests after a 12 hours activation period (figure 5A, B). Logically, IFN-γ production was decreased on activation of WT4 T cell clone by PD-L1 expressing T2 cell line loaded with the Melan-A_A27L_ peptide (figure 5A, left panel, dotted line), compared with the PD-L1 negative T2 cell line (solid line). In contrast, the reactivity of the PD-1^KO^ T cell clone was not affected by PD-L1 expression on peptide-loaded T2 cells (figure 5A, right panel). As expected, we obtained similar results with the wild-type and the PD-L1 expressing melanoma cell lines (figure 5B), namely that IFN-γ and IL-2 production were decreased on activation of WT4 T cell clone by PD-L1 expressing melanoma cells (left panel, dotted lines), compared with non-expressing ones (solid lines), whereas the expression of PD-L1 by melanoma cells did not alter IFN-γ and IL-2 production by KO6 T cell clone (figure 5B, right panel). Of note, we also observed that although not affected by PD-1/PD-L1 signaling, the KO6 T cell clone globally produced lower levels of IFN-γ and IL-2 than the WT4 T cell clone especially in response to melanoma cell lines, that could be a disadvantage for subsequent in vivo experiments. We therefore explored the ability of these two CTL clones to degranulate on 3-hour activation by melanoma cell lines expressing or not PD-L1. CD107a mobilization was decreased in WT4 T cell clone on activation with PD-L1 expressing cell line but remained unchanged for KO6 T cell clone (figure 5C). Globally, the percentages of T cells expressing CD107a were slightly higher for KO6 T cell clone, compared with the WT4. This result ensured that the two selected T cell clones have similar cytotoxic properties. Nonetheless, considering the differences observed for the production of IFN-γ and IL-2 by the two clones, we carried out their complete transcriptomic analysis before their use in vivo.

### Transcriptomic analysis

**Figure 6:**
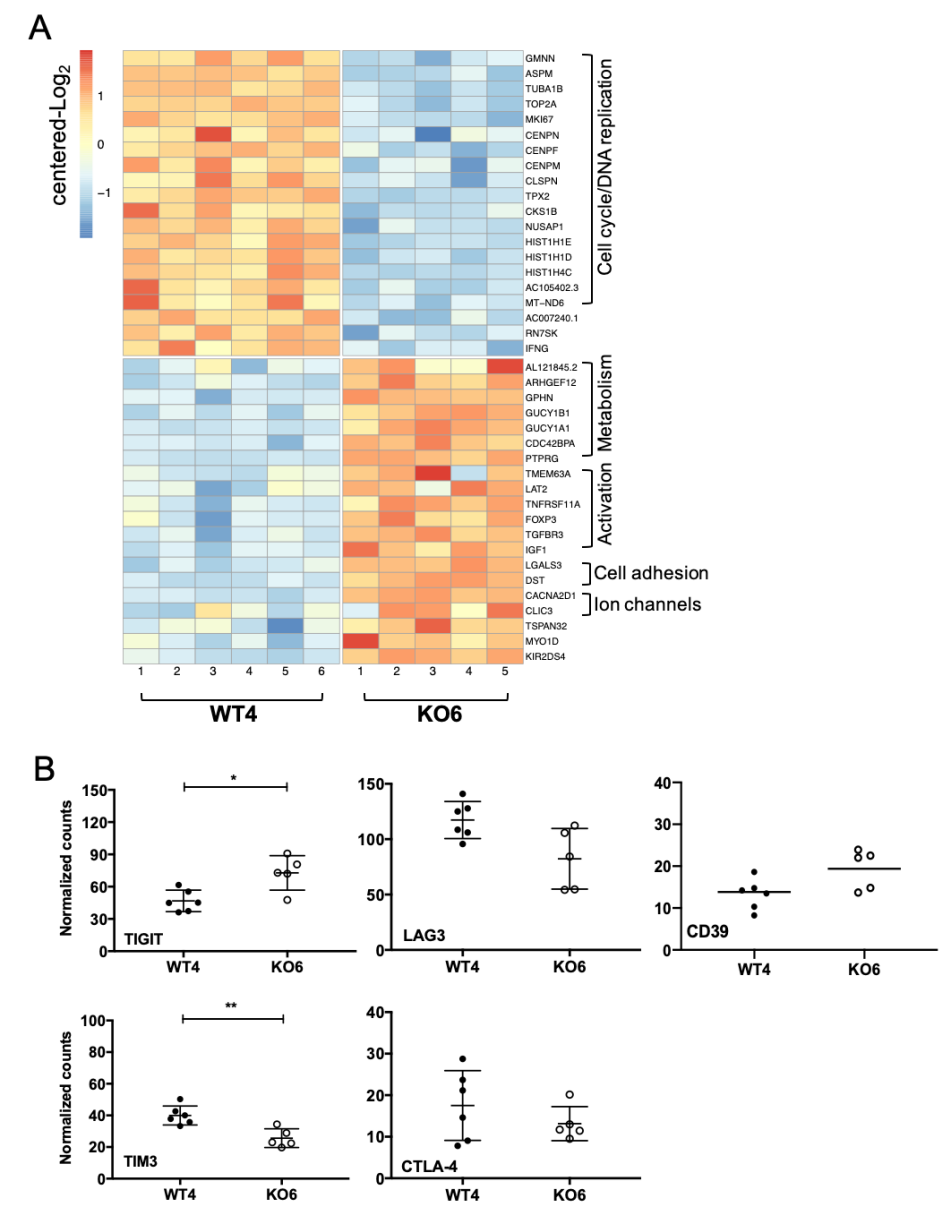
Transcriptomic comparison of WT4 and KO6 T cell clones and IC expression. (A) Heatmap reporting scale expression of the first 20 upregulated and downregulated genes (20 lowest adjusted p values, absolute log2 Fc >1) between WT4 and KO6 activated T cell clones. Genes are sorted by biologic functions. Each column represents a biologic replicate. (B) Comparative expression of IC coding genes between WT4 and KO6 T cell clones. **P<0.01 and *p<0.5 (Mann-Whitney non-parametric unpaired U test).

figure 6A illustrates the most significantly differentially expressed genes with at least an absolute log2 fold change of 1 (20 first upregulated and 20 first downregulated genes based on adjusted p value) between WT4 and KO6 T cell clones, after TCR activation. Each column represents a biologic replicate. Strikingly, the majority of downregulated genes in KO6 T cell clone were mainly related to proliferation and DNA replication. IFN-γ gene was also downregulated in KO6 T cell clone, confirming the results obtained by ELISA on activation with melanoma cell lines (figure 5B). Genes upregulated in KO6 T cell clone were more diverse with genes related to metabolism and cell signaling (Rho GTPases, protein kinases, ion channels…), T cell activation and cell adhesion. The comparison of all the TRBV3-1 T cell clones confirmed this picture, namely a decrease in the expression of proliferation and DNA replication-related genes in PD-1^KO^ T cell clones, paralleled with an increased metabolism (online supplementary figure S2A). We further focused on the expression of other ICs by KO6 T cell clone to evaluate any potential upregulation that would compensate the defect of PD-1 expression and explain the lower expression of IFN-γ by KO6 T cell clone. As shown on figure 6B, only TIGIT was significantly overexpressed by KO6 T cell clone (p=0.017; Mann-Whitney U test). This overexpression was consistent with the flow cytometry results illustrated by figure 4C, showing an increased expression level of TIGIT on KO6 T cell clone. LAG-3, CD39 and CTLA-4 expression were not significantly different between the two T cell clones, whereas TIM3 appeared underexpressed in KO6 T cell clone (p=0.0087; Mann-Whitney U test), also consistent with flow cytometry results (figure 4C). Globally, the transcriptomic comparison of all TRBV3-1 clonotypes confirmed the overexpression of TIGIT in PD-1^KO^ T cell clones (p=0.0037), whereas the expression of the other IC was not significantly different between WT and PD-1^KO^ T cell clones (online supplementary figure S2B).

10.1136/jitc-2019-000311.supp3 Supplementary data

### Antitumor activity of WT and PD-1^KO^ T cell clones

**Figure 7:**
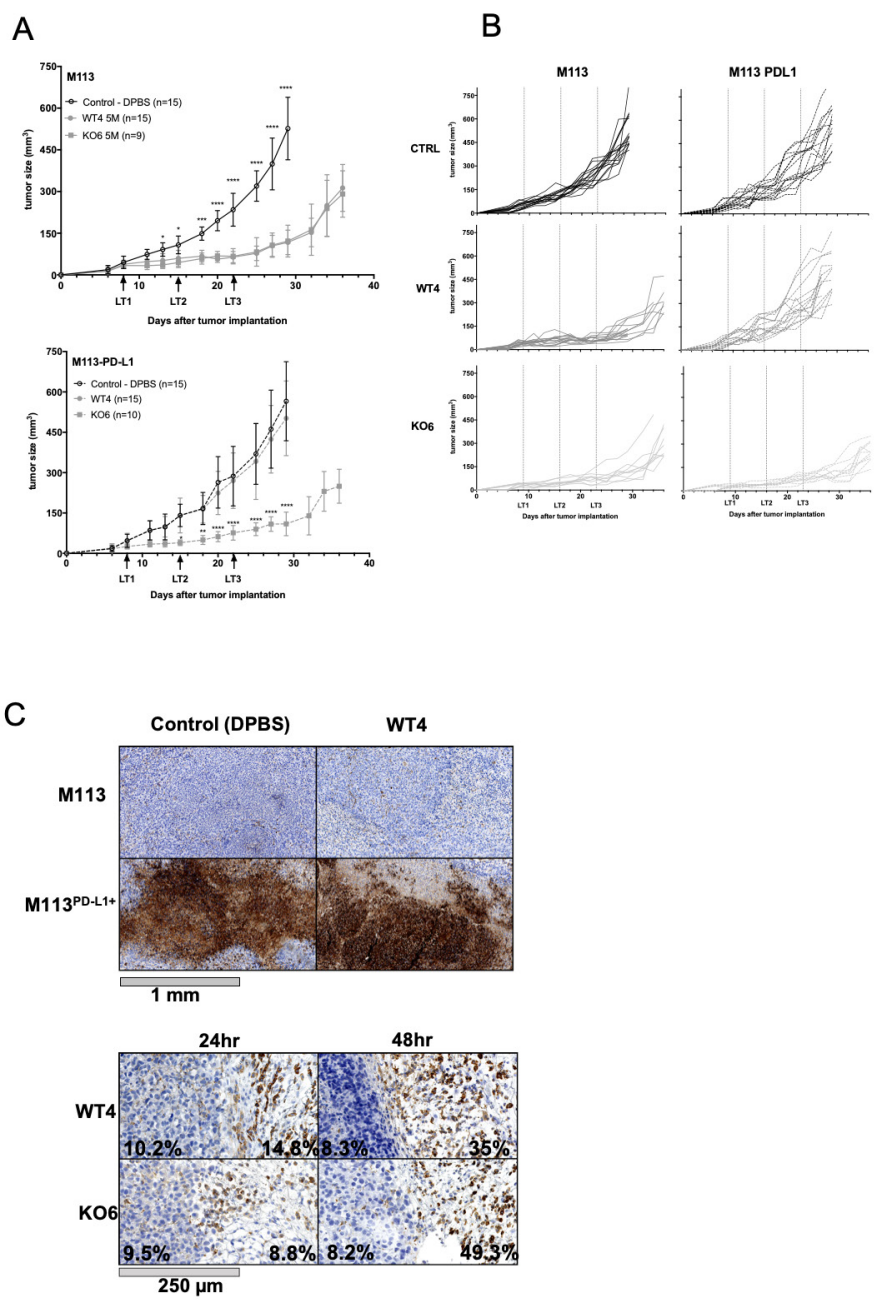
Antitumor efficiency of the adoptive transfer of WT4 and KO6 T cell clones. (A) Growth curves of engrafted M113 (up) and M113^PD-L1+^ melanoma cells (down), in NSG mice receiving intravenous injection of DPBS (empty circles) or intravenous injections of 5×10^6^ of WT4 (gray circles) or KO6 (gray squares) T cell clones. Intravenous injections are indicated with arrows. *P<0.05; **p<0.01; ***p<0.001 (two-way analysis of variance multiple comparisons). (B) Individual curves of in vivo growth of M113 (left) and M113^PD-L1+^ melanoma cells (right), in NSG mice receiving intravenous injection of DPBS (CTRL), or intravenous injections of 5×10^6^ of WT4 or KO6 T cell clones. (C) Upper panel: PD-L1 staining of M113 or M113^PD-L1+^ tumors from mice that have received either intravenous injection of DPBS (left) or WT4 T cell clone (right). Lower panel: CD3 staining of M113^PD-L1+^ tumors 24 hours or 48 hours after injection of either WT4 or KO6 T cell clones. Numbers indicate the percentages of CD3+ cells, within the tumor and in the peritumoral stroma (number of CD3+ cells relative to the total number of cells, quantified with the Qupath open source software).

Antitumor efficacy of WT and PD-1^KO^ CTL clones was assessed through their adoptive transfer in NSG mice, previously engrafted with a human melanoma cell line expressing PD-L1 (M113^PD-L1+^) or not (M113). Eight days after engraftment, the mice received either injection of DPBS or of 5×10^6^ T cells. These injections were repeated twice, on days 15 and 22. As illustrated by figure 7A, upper panel, and figure 7B, left panel, both WT and PD-1^KO^ T cell clones significantly delayed the growth of M113 melanoma tumors. A revival of tumor growth was observed 9 days after the last CTL injection (day 36). Conversely, the adoptive transfer of WT4 CTL clone was inefficient to delay the growth of PD-L1-expressing tumors (figure 7A, lower panel, and figure 7B, right middle panel), whereas the adoptive transfer of PD-1^KO^ CTL clones significantly delayed the growth of PD-L1-positive tumors, in a way comparable with that of PD-L1-negative tumors (figure 7A, lower panel, and figure 7B right lower panel). This result clearly showed that the adoptive transfer of PD-1-deficient melanoma-specific T cell clones resulted in a better tumor control than their wild-type counterpart.

On sacrifice, tumors were paraffin embedded for immunohistochemical analyses. PD-L1 expression analysis confirmed that PD-L1 was homogeneously and strongly expressed on M113^PD-L1+^ tumors and absent on wild-type tumors, with the exception of a few scattered islets (figure 7C, upper panel). We also documented the presence of CD3-positive T cells, in both peripheral and intratumor tissues 24 hours and 48 hours after injection of WT4 and KO6 T cell clones (figure 7C, lower panel). The percentages of CD3+ cells (number of CD3+ cells relative to the total number of cells, quantified by the Qupath software) were very similar after adoptive transfer of KO6 and WT4 T cell clones (figure 7C). However, a higher frequency of T cell clones were noted in the peritumoral stroma compared with intratumoral areas, 48 hours after ACT (figure 7C).

## Discussion

The development of genomic editing is a great opportunity to improve the antitumor functions of T cells for ACT purposes. In melanoma and other solid tumors, the therapeutic efficiency of ACT is weakened by the TME. Among the mechanisms that impede the effectiveness of injected tumor-specific T cells, the PD-1/PD-L1 axis is particularly important to target. Indeed, high avidity T cells or highly reactive T cells express high levels of PD-1^13 37 38^ which, on binding with its ligand expressed by tumor cells or other cells from the TME, results in decreased TCR signaling and impaired effector functions. The adoptive transfer of PD-1-deficient tumor-specific T cells, engineered through CRISPR/Cas9 editing, have a major interest in increasing the clinical efficacy of adoptive transfer approaches. Until now, CRISPR/Cas9 editing had been successfully used to disrupt *PDCD1* gene in CAR-T cells targeting either CD19,^20^ mesothelin^22^ or HCC.^21^ However, although the feasibility of *PDCD1* editing had been shown in human primary CTL,^24 25^ the feasibility to disrupt *PDCD1* gene in CD8^+^ effector/memory T cells specific for tumor antigens was still lacking. Here we showed the efficiency of *PDCD1* editing in melanoma-specific CTL produced according to a procedure currently used in a clinical trial in metastatic melanoma patients (MELSORT trial, NCT02424916). All the edited T cell clones were edited on a single *PDCD1* allele, mainly through NEHJ pathway (8/9 CTL clones). The insertion of the HDR template was observed in only one T cell clone (KO4). It is well admitted that the efficiency of HDR crucially depends on cell cycle phases and is variable according to the cell types. Our experimental conditions were probably not optimal to favor this pathway compared with NHEJ, a fast mechanism active throughout the cell cycle.^39^ Although the recombination event only occurred on a single allele, this led either to a drastic decrease in the expression of PD-1 or to its complete absence of expression on activated T cell clones (figure 2). As we could not observe any significant differences of *PDCD1* expression measured by qPCR in WT and PD-1^KO^ T cell clones (figure 3C), and as *PDCD1* does not belong to genes subject to random monoallelic expression,^40^ the mechanisms (probably translational or post-translational) explaining the total absence of PD-1 expression in these T cell clones edited on a single *PDCD1* allele remain to be explored.

The ultimate goal of our study was to compare the antitumor functions of melanoma-specific CTL only differing by PD-1 expression; therefore, we selected a pair of CTL clones expressing the same TCR and expressing or not PD-1 (WT4 and KO6). We confirmed the absence of PD-1 expression by the KO6 T cell clone on stimulation both with anti-CD3 or melanoma cell lines, by flow cytometry and western blot. We also validated that these two T cell clones exhibited the same functional avidity for their cognate peptide (figure 4B) and that the reactivity of the PD-1-deficient clonotype was not impaired by the presence of PD-L1 on peptide-loaded T2 cell line or melanoma cells in vitro (figure 5). Unexpectedly, we also observed that although not affected by PD-1/PD-L1 signaling, the KO6 T cell clone globally produced lower levels of IFN-γ and IL-2 than the WT4 T cell clone especially in response to melanoma cell lines. This difference could be due to the overexpression of other IC by this PD-1^KO^ T cell clone, whose binding to their ligands expressed by tumor cells could lead to a decreased reactivity. In this context, with the exception of TIGIT (overexpressed on the KO6 clone) and Tim-3 (underexpressed on the activated KO6 clone), we did not observe any major differences in expression of the costimulatory molecules or other ICP between the two clones (figure 4C, D). The significant overexpression of TIGIT by PD-1^KO^ T cell clones was subsequently confirmed by transcriptomic analysis on the WT4 and KO6 T cell clone, and on the other PD-1^KO^ T cell clones, expressing the same TCR (figure 6B and online supplementary figure S2B). This feature may participate to the lower production of IFN-γ and IL-2 by the KO6 clone in response to melanoma lines, which spontaneously express the two main TIGIT ligands, CD155 and CD112, in contrast to T2 cell lines (data not shown). Nevertheless, transcriptomic analysis also revealed that the IFN-γ gene was significantly less expressed in the KO6 T cell clone, activated by an anti-CD3 antibody, compared with the WT4 T cell clone (figure 6A), as well as in PD-1^KO^ compared with wild-type T cell clones expressing the same TCR (online supplementary figure S2A). This suggests that the decreased expression of IFN-γ by PD-1^KO^ T cell clones is also due to an intrinsic modification, probably caused by the absence of PD-1 expression by these clones and leading to a phenotype similar to that of exhausted T lymphocytes, although these lymphocytes do not overexpress the markers commonly found on exhausted T lymphocytes (CD39, Tim-3…). The mechanisms explaining this downregulation of IFN-γ therefore remain to be explored. The majority of other downregulated genes were related to cell cycle and DNA replication, suggesting that PD-1 deletion also impaired replicative properties of CTL clones, while upregulated genes in PD-1^KO^ T cell clones were almost all related to metabolism and cell signaling. Despite these features, the antitumor properties of KO6 T cell clone were significantly increased in vivo, as the adoptive transfer of KO6 T cell clone significantly delayed the growth of human melanoma tumors expressing PD-L1, compared with the adoptive transfer of WT4 CTL clone. These findings suggest that, in this setting, the recovery of optimal effector functions such as lytic properties are more crucial than the proliferative capacities of infused T cells. Our results are consistent with a previous study demonstrating, in a mouse model, that the genetic absence of PD-1 led to the accumulation of more cytotoxic exhausted CD8^+^ T cells.^41^ Furthermore, the expression of metabolism-related genes, increased in *PDCD1*-edited T cell clones, could also give them an advantage in the TME.

Interestingly, although PD-L1 expression on tumor cells was artificially induced by transfection, PD-L1 expression profile observed on tumors (figure 7C, upper panel) was similar to that of human tumors constitutively expressing PD-L1 through genetic alterations, leading to an homogeneous PD-L1 expression on cancer cells.^42^ Furthermore, despite their lower proliferation potential, we documented the presence of KO6 T cell clone within tumors to the same extend as WT4 T cell clone, as long as 48 hours after injection (figure 7C, lower panel), with a higher frequency of T cell clones in the peritumoral areas at this time-point. This last result suggests that, although infused T cells migrate on the tumor site, T cell infiltration into the tumor is still too limited for optimal therapeutic efficacy and that the use of a therapeutic combination to increase tumor vascularization or modify tumor structure could promote more massive infiltration and increase therapeutic benefit.

Finally, the adoptive transfer of such modified T cells with optimized effector functions in an immunocompetent host could also induce additional immune responses, through epitope spreading, shown to be associated with clinical responses.^10 43^ The induction of this phenomenon does not require long-term survival of effector T cells, but rather an immediate increased efficiency in the TME.

Finally, the efficiency of effector T cells within the TME could be further improved through the deletion of a combination of IC, as previously documented for PD-1 and LAG-3,^44^ PD-1 and Tim-3^45^ and PD-1 and TIGIT^46^ for antiviral responses. In each of these studies, the simultaneous inhibition of the two pathways synergistically reversed T cell exhaustion and improved T cell functions. In our model, TIGIT was the only IC significantly overexpressed in PD-1^KO^ T cell clones, suggesting compensatory mechanisms between these two IC, consistent with the synergy of their signalization pathways.^47^ This suggests that, in this setting, coediting *PDCD1* and *TIGIT* genes could further enhance the antitumor properties of these CTL clones.

## Conclusions

In conclusion, this study demonstrates the feasibility of *PDCD1* editing in effector memory CTL, specific for melanoma antigens and clearly showed their superior efficiency in delaying the growth of PD-L1-positive melanoma tumors. The use of such lymphocytes for ACT purposes, associated with other approaches modulating the TME, in order to favor the infiltration of therapeutic T cells in the tumors, would greatly enhance the efficacy of antitumor immunotherapy treatments.
