# Supplementary material for: Increased antitumor efficacy of PD-1-deficient melanoma-specific human lymphocytes
Source: J Immunother Cancer. 2020 Jan 29;8(1):e000311. doi: 10.1136/jitc-2019-000311 (PMC7057432; doi:10.1136/jitc-2019-000311)
Supplement: Supplementary data [file jitc-2019-000311supp001.pdf]

Table S1 : Off-target genes of *PDCD1*-specific sgRNA

	Gene name	cfd off target score	F-PCR primer (5'-3')	R-PCR primer (5'-3')	Amplicon size bp	Expected T7E1 fragment size	T7E1 results
***PDCD1* in target**	**PDCD1**		GCCACAGCAGTGAGCAGAGA	GGGGCTCATCCCATCCTTAG	**905**	**548/357**	**548/337***
PD1-OT1	TEX29-RP11-65D24.2 (intergenic)	0,46	ACCCCTCTTAGTACGCAGGA	GCGTGCCCCATTACATTTCG	773	470/303	480/280**
PD1-OT2	NFIX-AC007787.2 (intergenic)	0,40	TGTGGCTGTTCCAGAGGTTAC	TGTGCTCTTAGCTGCTTCCC	592	516/76	No heteroduplexes
PD1-OT3	RP11-66B24.5-AC087762.1 (intergenic)	0,30	AAGGGGGTCCAAGTTCCTCT	TCAGCCAGACTTCTTCCCCT	599	379/220	500/300**
PD1-OT4	THADA (intron)	0,29	GAGGCCAGACCACTGACTTG	GGCTTCATGCTCCAGACCTT	669	444/225	450/250**
PD1-OT5	TUBGCP3-RP11-88E10.4 (intergenic)	0,25	CGCCATTTTAACGGAACCCG	CTCGGCAGGTTTCCTGTTCT	563	438/125	No heteroduplexes
PD1-OT16	CH507-42P11.5 = LINC01669 (intron)	0,17	TTCAGAAAATGGCCAAGCCCT	GAAGCCTGTGAAAACACTGGG	638	412/226	No heteroduplexes
PD1-OT18	FNBP1 (intron)	0,17	CTCGTCGTCAAAATCCGTGG	GGCCTTAGGTTGGAGTCTCT	560	360/200	No heteroduplexes
PD1-OT19	C20orf27 (intron)	0,13	CTCGCTCCCCACTTCTCAAT	CGGGATTGTTCCTGGCTGAA	638	433/205	No heteroduplexes
PD1-OT30	SNHG1/SNORD25 (exon)	0,08	GCCCTGACATTTGTTGCGTA	TGGTAAGTGGCTTCGTGGTC	609	465/139	No heteroduplexes

*Fragments detected in *PDCD1*-edited T cell population

** Fragments detected both in WT and *PDCD1*-edited T cell population

Table S2 : CDR3 alpha and beta sequences of WT and *PDCD1* edited melanoma-specific T cell clones

T cell clone	TRBV	CDR3 beta	TRBJ	TRAV	CDR3 alpha	TRAJ	Event on PDCD1 gene
**WT T cell clones**							
WT1	TRBV28	CASMRTGLGIEQYFG	TRBJ2-7	TRAV12-2	CAGNTGGFKTIFG	TRAJ9	
WT2	TRBV6-1	CASSDTGLGQPQHFG	TRBJ1-5	TRAV12-2	CAVNWGQNFVFG	TRAJ26	
WT3	TRBV6-2	CASSRGVGQPQHFG	TRBJ1-5	TRAV12-2	CAVSSDGQKLLFA	TRAJ16	
WT4	TRBV3-1	CASSPLGTPSYEQYFG	TRBJ2-7	TRAV12-2	CAVPFGNEKLTFG	TRAJ48	
WT5							
***PDCD1*-edited T cell clones**							
KO2	TRBV3-1	CASSPLGTPSYEQYFG	TRBJ2-7	TRAV12-2	CAVPFGNEKLTFG	TRAJ48	53-nt deletion : ATG loss
KO6							
KO11							
KO1							Single nucleotide deletion : Frame-shift and STOP end Ex1
KO5							7-nt deletion : Frame-shift and STOP end Ex1
KO7	TRBV6-2	CASTQGMGQPQHFG	TRBJ1-5	TRAV12-2	CALGQAGTALIFG	TRAJ15	10 nt-deletion : Frame-shift and STOP end Ex1
KO13							
KO4							Insertion of HDR template : Frame shift and STOP end Ex1
KO10	TRBV28	CASRPQGLGQPQHFG	TRBJ1-5	TRAV12-1	CVTPNDYKLSFG	TRAJ20	25-nt deletion : ATG loss
