# Supplementary material for: Increased antitumor efficacy of PD-1-deficient melanoma-specific human lymphocytes
Source: J Immunother Cancer. 2020 Jan 29;8(1):e000311. doi: 10.1136/jitc-2019-000311 (PMC7057432; doi:10.1136/jitc-2019-000311)
Supplement: Supplementary data [file jitc-2019-000311supp002.pdf]

Figure S1

A

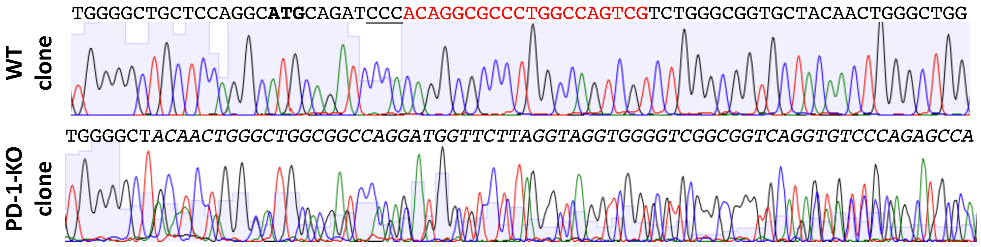

B

```
WT PD-1 (n=5)
GCGGCACTCTGGTGGGGCTGCTCCAGGCATGCAGATCCCACAGGCGCCCTGGCCAGTCGTCTGGGCGGTGCTACAACTGGGCTGGCGGCCAGGATGG
KO2;KO6;KO11 (TRBV3-1)
GCGGCACTCTGGTGGGGCT-----------------------------------------------------ACAACTGGGCTGGCGGCCAGGATGG
KO1 (TRBV3-1)
GCGGCACTCTGGTGGGGCTGCTCCAGGCATGCAGATCCCAC-GGCGCCCTGGCCAGTCGTCTGGGCGGTGCTACAACTGGGCTGGCGGCCAGGATGG
KO5 (TRBV3-1)
GCGGCACTCTGGTGGGGCTGCTCCAGGCATGCAGATCCCACAG-CG------CCAGTCGTCTGGGCGGTGCTACAACTGGGCTGGCGGCCAGGATGG
KO7;KO13 (TRBV6-2)
GCGGCACTCTGGTGGGGCTGCTCCAGGCATGCAGATCCC----------TGGCCAGTCGTCTGGGCGGTGCTACAACTGGGCTGGCGGCCAGGATGG
KO4 (TRBV6-2)
GCGGCACTCTGGTGGGGCTGCTCCAGGCATGCAGATAATGAAAGCTT-CTGGCCAGTCGTCTGGGCGGTGCTACAACTGGGCTGGCCGCCAGGATGG
KO10 (TRBV28)
GCGGCACTCTGGTGGGGCTGCTCCAGGC-------------------------CAGTCGTCTGGGCGGTGCTACAACTGGGCTGGCGGCCAGGATGG
```
