# Supplementary figures and images for: Increased antitumor efficacy of PD-1-deficient melanoma-specific human lymphocytes
Source: J Immunother Cancer. 2020 Jan 29;8(1):e000311. doi: 10.1136/jitc-2019-000311 (PMC7057432; doi:10.1136/jitc-2019-000311)

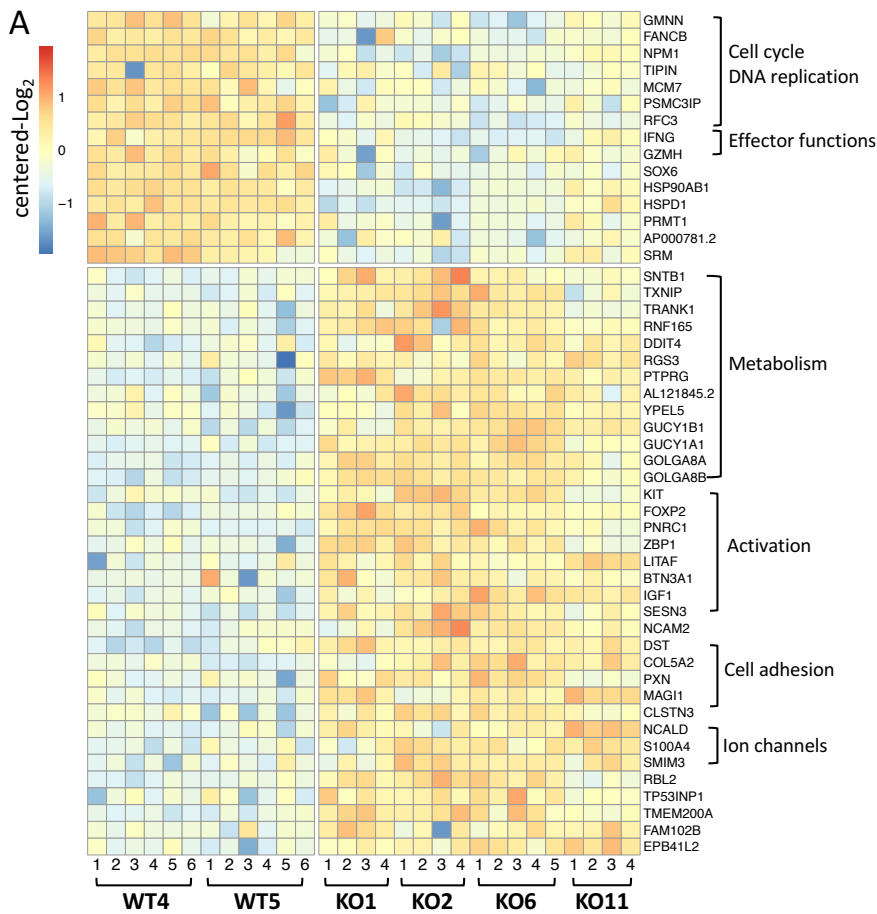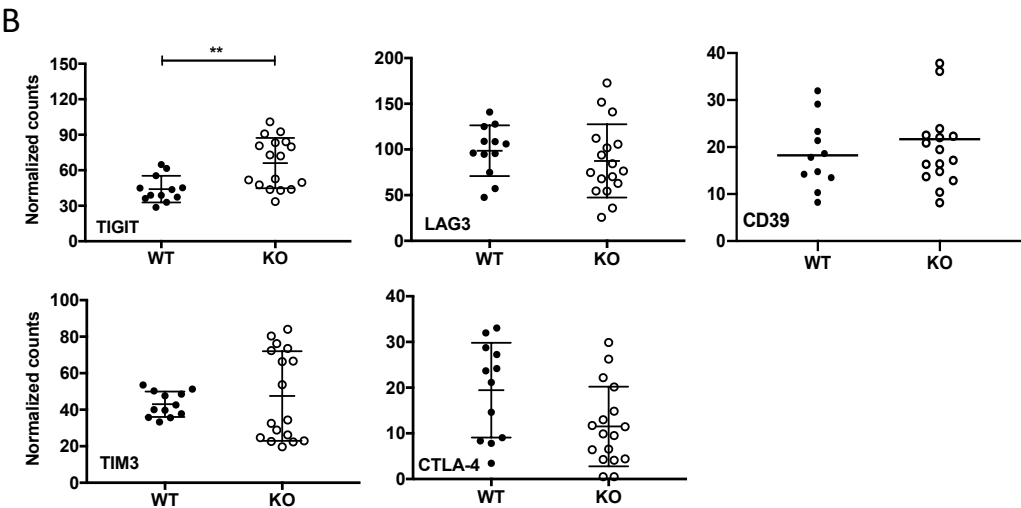

Supplement: Supplementary data [file jitc-2019-000311supp003.pdf]
